# Response of Cd, Zn Translocation and Distribution to Organic Acids Heterogeneity in *Brassica juncea* L.

**DOI:** 10.3390/plants12030479

**Published:** 2023-01-19

**Authors:** Yumeng Liao, Zuran Li, Zhichen Yang, Jixiu Wang, Bo Li, Yanqun Zu

**Affiliations:** 1 College of Resources and Environment, Yunnan Agricultural University, Kunming 650201, China; 2 College of Horticulture and Landscape, Yunnan Agricultural University, Kunming 650201, China

**Keywords:** heavy metal, *Brassica juncea* L., xylem, translocation factor

## Abstract

In order to investigate the translocation, distribution, and organic acid heterogeneity characteristics in *Brassica juncea* L., a pot experiment with the exogenous application of Cd and Zn was conducted to analyze the effects of Cd, Zn, and organic acid contents and heterogeneity on the translocation and distribution of Cd and Zn. The results showed that the Cd and Zn contents of *B. juncea* were mainly accumulated in the roots. The Cd content in the symplast sap was 127.66–146.50% higher than that in the apoplast sap, while the opposite was true for Zn. The distribution of Cd in xylem sap occupied 64.60% under 20 mg kg^−1^ Cd treatment, and Zn in xylem sap occupied 60.14% under 100 mg kg^−1^ Zn treatment. The Cd was predominantly distributed in the vacuole, but the Zn was predominantly distributed in the cell walls. In addition, oxalic and malic acids were present in high concentrations in *B. juncea*. In the vacuole, correlation analysis showed that the contents of Cd were negatively correlated with the contents of oxalic acid and succinic acid, and the contents of Zn were positively correlated with the contents of malic acid and acetic acid. The contents of Cd and Zn were negatively related to the contents of oxalic acid and citric acid in xylem sap. Therefore, Cd in *B. juncea* was mainly absorbed through the symplast pathway, and Zn was mainly absorbed through the apoplast pathway, and then Cd and Zn were distributed in the vacuole and cell walls. The Cd and Zn in *B. juncea* are transferred upward through the xylem and promoted by oxalic acid, malic acid, and citric acid.

## 1. Introduction

Cadmium (Cd) is a toxic heavy metal that affects the uptake of mineral nutrients in plants, causes stunted growth and yellowing of leaves, and inhibits photosynthesis [1,2,3]. Zinc (Zn) is one of the essential micronutrients for plant growth, forming metalloenzymes, proteins, and transcription factors in plants and thus functioning of photosynthesis, hormone regulation, and zinc-iron transport protein (ZIP) synthesis [4,5]. However, excess Zn can be detrimental to plants by affecting physiological and biochemical processes [6,7].

In recent times, physical, chemical, and biological approaches have been widely applied in the remediation of heavy metal soil. Among these strategies, phytoremediation has been supported by many scholars due to its green and sustainable advantages [8,9,10]. Previous studies found that *Brassica juncea* L. are tolerance and enrichment for Cd, Zn, Ni, and Cr, making it a model plant for studying phytoremediation technology [11,12,13]. Therefore, *B. juncea* is considered to have great remediation potential in polluted soil. However, most studies have concentrated on the accumulation of Cd and Zn instead of the translocation of Cd and Zn in plants [7,14]. Moreover, the coping mechanism of organic acids in *B. juncea* under Cd and Zn stress is still unclear.

A previous study found two pathways for plants to absorb Cd and Zn, including the apoplast (the cell wall and the space between cells) and symplast pathways (the intercellular filaments between cells) [15]. However, the present study does not address the question of whether plants are dominated by symplast or apoplast uptake. Once loaded in the xylem, Cd and Zn can be transported upward to the leaves with water and downward to other parts with nutrients through the phloem [16,17]. The Cd and Zn absorbed by roots can be transported to plant tissues over long distances through xylem and phloem [18,19]. Cd and Zn can be bound to some substances in plant cells and selectively distributed in subcellular sites [19]. It has been found that Cd and Zn were connected by the cell wall or entered the vacuole via transport proteins, thus diminishing toxicity [20,21]. Cd and Zn can combine with citric acid in the xylem sap, which is later transported to shoots [22,23]. Under different concentrations of Cd stress, oxalic acid and citric acid secreted by plants can be complexed with Cd to form low- or non-toxic compounds [24]. Therefore, the toxicity and mobility of Cd and Zn in plants are related to their subcellular distribution [25]. However, apart from these studies, few studies have analyzed the association between organic acids and Cd and Zn in xylem sap.

As a result, we hypothesized that the organic acid heterogeneity of Cd and Zn in *B. juncea* is not identical. In order to test this hypothesis, we conducted pot experiments using Cd and Zn solutions as exogenous additions. The specific objectives were to (i) examine whether Cd and Zn are more distributed in the symplast or the apoplast and whether their accumulation in shoots depends on the upward transport in the xylem, and (ii) check whether oxalic and citric acids can affect the accumulation and translocation of Cd and Zn. The results will provide a scientific basis for the translocation and distribution of heavy metals in plants and the phytoremediation of soil heavy metal contamination.

## 2. Results

### 2.1. Plant Biomass and Cd and Zn Contents

The *B. juncea* biomass decreased under Cd treatment, while shoot and root biomass decreased by 29.29% and 45.66% under 300 mg kg^−1^ Zn treatment, respectively (Table 1). Compared to the control, Cd contents in shoots increased by 565.83% and 674.53% under 20 mg kg^−1^ Cd and 40 mg kg^−1^ Cd treatments, respectively. In addition, with increased Cd treatment concentrations, the Cd contents in roots gradually increased 17.5 and 48.23 times that of the control, respectively. Further, with the increase in Zn treatment concentrations, the Zn contents in shoots gradually increased by 134.78% and 145.09%, respectively. With the increase in Zn treatment concentrations, the Zn contents in roots also gradually increased by 252.48% and 597.78%, respectively. The transfer factor decreased with the increase in Cd and Zn treatment concentrations.

### 2.2. Contents of Cd and Zn in Sap Associated with Horizontal and Vertical Translocation Pathways of B. juncea

#### 2.2.1. The Contents of Cd and Zn in the Sap Associated with the Horizontal Translocation Pathway of *B. juncea*

**Figure 1:**
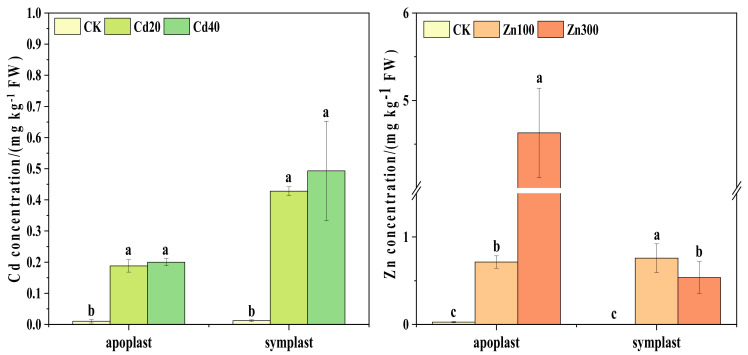
Cd and Zn contents in apoplast and symplast of *B. juncea* roots. The different lowercase letters indicate at the *p ≤* 0.05 level of significance under different Cd and Zn treatment concentrations. The legend indicates the treatment concentrations/(mg kg^−1^). The same as below.

Under Cd treatments, the Cd contents of both apoplast and symplast saps in *B. juncea* roots were significantly higher than CK, and the Cd contents of symplast sap were higher than those of apoplast sap (Figure 1). Under 20 mg kg^−1^ and 40 mg kg^−1^ of Cd treatments, Cd contents in apoplast sap were 18.80 and 20 times higher than CK, respectively. The Cd contents of symplast sap were 35.67 and 41.08 times those of CK, respectively.

The Zn content in apoplast sap showed a significant increase with increasing Zn treatment concentrations. In addition, the Zn contents in apoplast sap were higher than those in symplast sap. Further, the Zn content in apoplast sap reached 4.63 mg kg^−1^ under 300 mg kg^−1^ Zn treatment, which was 6.48 times higher than that under 100 mg kg^−1^ Zn treatment.

#### 2.2.2. Contents of Cd and Zn in the Sap Associated with the Vertical Translocation Pathway of *B. juncea*

**Figure 2:**
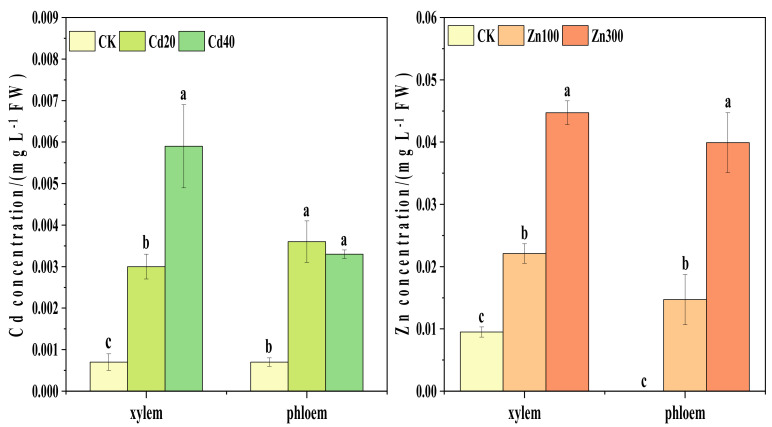
Cd and Zn contents in xylem and phloem sap of *B. juncea*. The different lowercase letters indicate at the *p ≤* 0.05 level of significance under different Cd and Zn treatment concentrations.

With the increase in Cd treatment concentration, the Cd content in xylem sap showed a significant increasing trend with a 309.09% and 709.09% increase, respectively (Figure 2). Under 40 mg kg^−1^ Cd treatment, the Cd content in xylem sap reached 0.006 mg L^−1^, which was 97.78% higher than that under 20 mg kg^−1^ Cd treatment. Compared to CK, the Cd contents in phloem sap under 20 mg kg^−1^ and 40 mg kg^−1^ Cd treatments increased by 407.14% and 364.29%, respectively.

The Zn contents in xylem and phloem sap showed a significant increase with the increases in Zn treatment concentrations. Under 100 mg kg^−1^ Zn treatments, the Zn contents in xylem sap were 50.85% higher than those in phloem sap. Compared to CK, Zn contents in xylem sap under 100 mg kg^−1^ and 300 mg kg^−1^ Zn treatments increased by 133.25% and 371.24%, respectively. The contents of Cd and Zn in xylem sap were higher than those in phloem sap.

### 2.3. Subcellular Distribution of Cd and Zn in Roots, Stems and Leaves of B. juncea

#### 2.3.1. Subcellular Distribution of Cd in Roots, Stems, and Leaves of *B. juncea*

**Figure 3:**
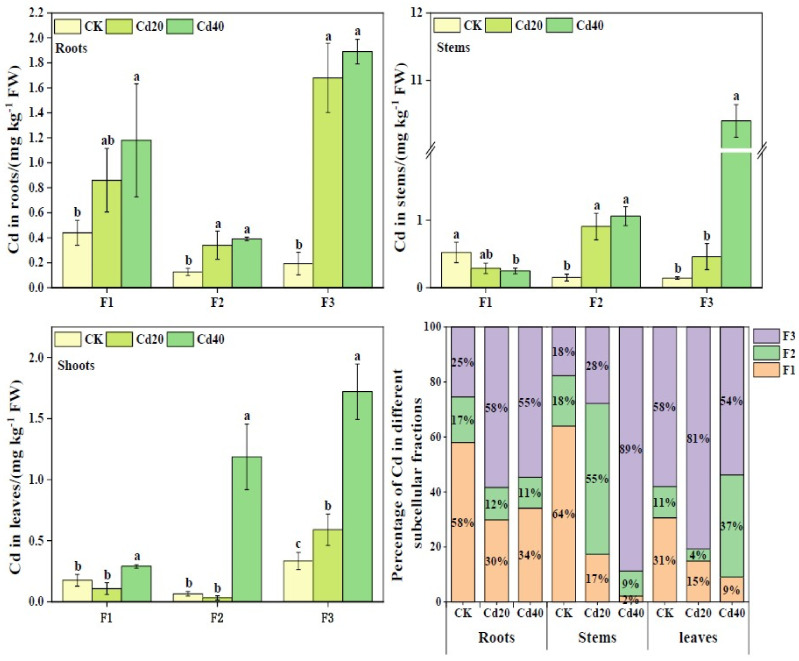
Subcellular distribution and percentage of Cd in roots, stems, and leaves of *B*. *juncea*. The legend shows different concentrations of Cd treatment (unit: mg kg^−1^). Different lowercase letters indicate that the difference reaches the *p ≤* 0.05 level of significance under different Cd treatment levels. F1: cell wall fraction; F2: organelle fraction; F3: vacuole fraction. The same as below.

The Cd contents of the cell wall fraction, organelle fraction, and vacuole fraction in *B. juncea* roots gradually increased with increasing Cd treatment concentrations, ranging from 0.44–1.18 mg kg^−1^, 0.13–0.39 mg kg^−1^, and 0.19–1.89 mg kg^−1^, respectively (Figure 3). The Cd contents of the cell wall fraction, organelle fraction, and vacuole fraction increased by 167.86%, 209.03%, and 877.86% under a 40 mg kg^−1^ Cd treatment concentration compared to CK. In addition, the Cd contents of the cell wall fraction, organelle fraction, and vacuole fraction in the leaves of *B. juncea* ranged from 0.18–0.29 mg kg^−1^, 0.03–1.19 mg kg^−1^, and 0.33–1.72 mg kg^−1^, respectively, as the concentration of Cd treatment increased. The cell wall fraction and vacuole fraction Cd content of leaves under 40 mg/kg Cd treatment concentration increased by 64.23% and 414.27%, respectively, compared to CK.

The Cd contents in *B. juncea* showed vacuoles > cell walls > organelles. Under Cd treatments, the percentage of Cd content in the vacuoles of roots and leaves was 55–58% and 54–81%, respectively, and the percentage of Cd content in the cell wall was 30–34% and 9–15%, respectively. Compared with CK, the percentage of Cd content in the cell wall fraction decreased under Cd treatments, whereas the percentage of Cd content in the vacuole fraction increased.

#### 2.3.2. Subcellular Distribution of Zn in Roots, Stems, and Leaves of *B. juncea*

**Figure 4:**
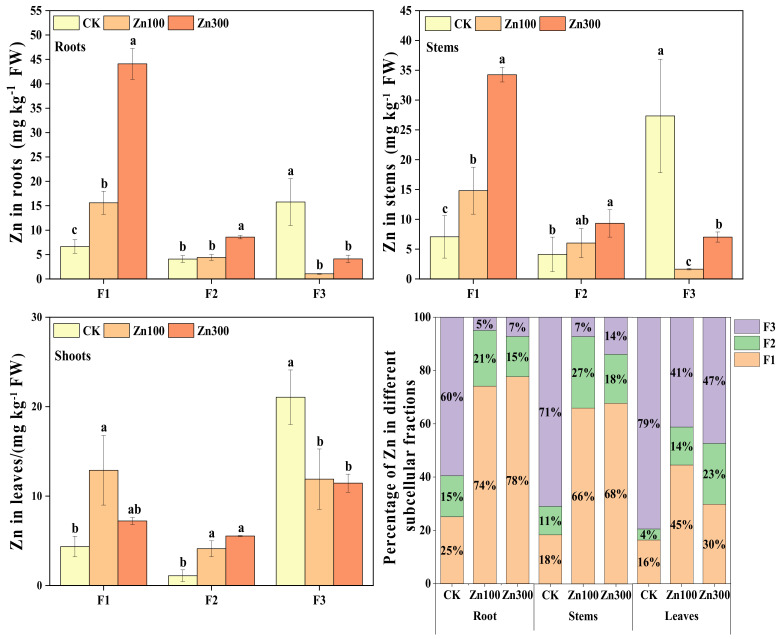
Subcellular distribution and percentage of Zn in roots, stems and leaves of *B. juncea*. The legend shows different concentrations of Zn treatment (unit: mg kg^−1^). Different lowercase letters indicate that the difference reaches at the *p ≤* 0.05 level of significance under different Zn treatment levels.

The Zn contents of the cell wall and organelle fractions in *B. juncea* roots gradually increased with increasing Zn treatment concentrations, ranging from 6.65–44.08 mg kg^−1^ and 4.08–8.56 mg kg^−1^, respectively (Figure 4). In addition, the Zn contents of the vacuole fraction ranged from 1.04–15.78 mg kg^−1^. The Zn contents of the cell wall and organelle fractions increased by 562.96% and 109.80%, respectively, and the Zn contents of the vacuole fraction decreased by 73.96% under 300 mg kg^−1^ Zn treatment compared with CK. Further, the Zn contents of the cell wall fraction and organelle fraction in *B. juncea* gradually increased with increasing Zn treatment concentration, ranging from 7.06–34.25 mg kg^−1^ and 4.12–9.32 mg kg^−1^, respectively, and the Zn content of the vacuole fraction ranged from 1.64–27.34 mg kg^−1^. The Zn content of the cell wall and organelle fraction increased by 385.17% and 126.21%, respectively, and the Zn content of the vacuole fractions decreased by 74.32% under 300 mg kg^−1^ Zn treatment compared with CK. Finally, Zn contents of the cell wall and organelle fractions increased by 195.57% and 273.94%, respectively, and Zn contents of the vacuole fraction decreased by 43.49%.

The Zn contents in *B. juncea* showed cell wall > vacuole > organelle. Under Zn treatments, the percentages of Zn content in the cell wall fractions of roots, stems, and leaves were 74–78%, 66–68%, and 30–45%, respectively, and the percentage of Zn content in the vacuole of leaves was 41–47%. Compared with CK, the percentage of Zn content in cell wall fractions increased under Zn treatments, while the percentage of Zn content in the vacuole fraction decreased.

### 2.4. Organic Acid Heterogeneity in B. juncea under Cd and Zn Treatments

#### 2.4.1. Organic Acid Heterogeneity in *B. juncea* under Cd Treatments

**Figure 5:**
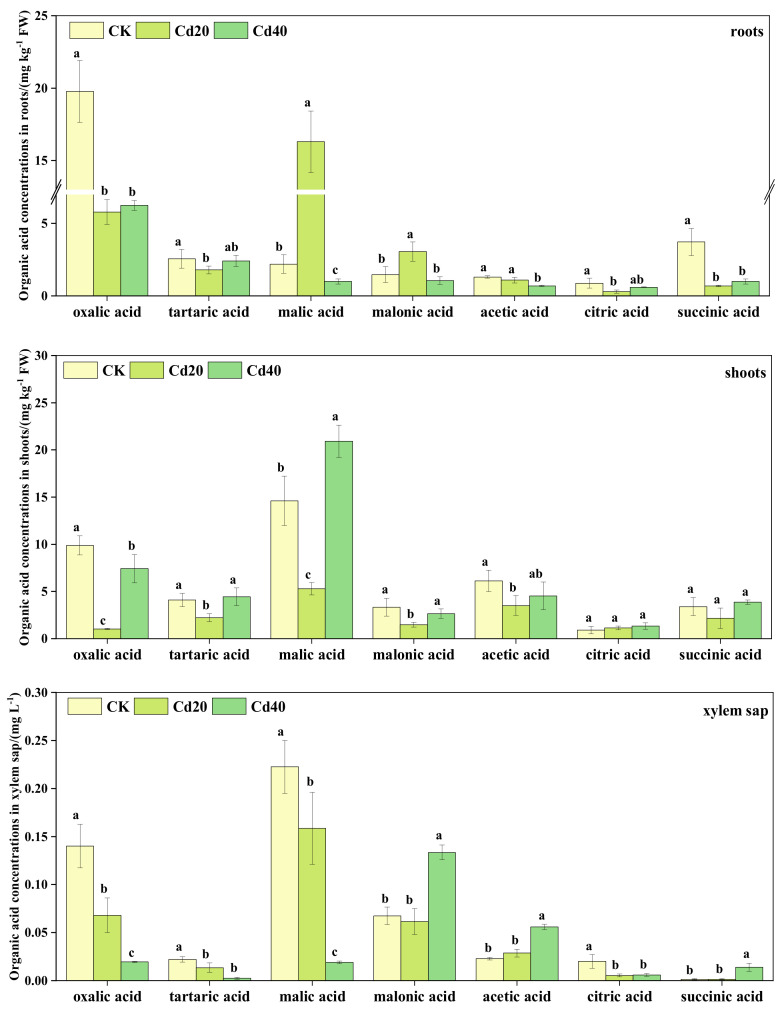
Organic acid contents in roots, shoots and xylem sap of *B. juncea* under Cd treatments. The legend shows different concentrations of Cd treatment (unit: mg kg^−1^). Different lowercase letters indicate that the difference reaches at the *p ≤* 0.05 level of significance under different Cd treatment levels.

The organic acid contents in roots, shoots, and xylem sap of *B. juncea* under Cd treatment varied (Figure 5). The organic acid in *B. juncea* roots was mainly oxalic acid and malic acid, both of which accounted for 19.95–61.99% and 6.88–56.20% of the total organic acids in the roots, respectively. Oxalic acid showed a decreasing trend with increasing Cd treatment concentrations, and malic acid first increased and then decreased. The organic acids on shoots were mainly oxalic acid, malic acid, and acetic acid, which accounted for 6.12–23.34%, 31.43–46.32%, and 10.03–20.87% of the total organic acids, respectively, and oxalic acid showed that roots > shoots, while malic acid was the opposite. The organic acids in xylem sap mainly consisted of oxalic acid, malic acid, and malonic acid, which accounted for 7.78–22.20%, 7.58–47.03%, and 13.60–53.27% of the total organic acids, respectively. Compared with CK, the contents of oxalic acid in xylem sap decreased by 51.43%, 86.07%, and malic acid in xylem sap decreased by 28.76%, 91.46%, under 20 mg kg^−1^ and 40 mg kg^−1^ Cd treatment concentrations, respectively. The contents of malonic acid increased by 97.78% compared to CK.

#### 2.4.2. Organic Acid Heterogeneity in *B. juncea* under Zn Treatments

**Figure 6:**
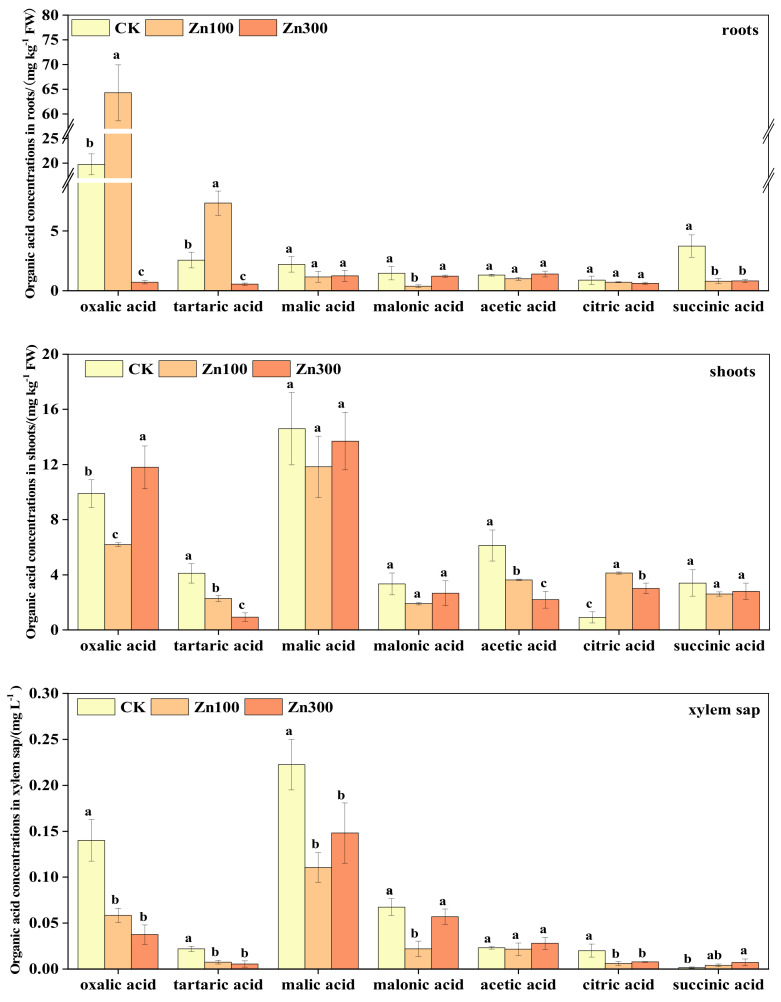
Organic acid content in roots, shoots and xylem sap of *B. juncea* under Zn treatments. The legend shows different concentrations of Zn treatment (unit: mg kg^−1^). The different lowercase letters indicate that the difference reaches at the *p ≤* 0.05 level of significance under different Zn treatment levels.

Figure 6 shows the changes in organic acid content in *B. juncea* under Zn treatment. Oxalic acid and tartaric acid were mainly in the roots of *B. juncea*, accounting for 10.88–84.97% and 8.39–9.66% of the total organic acids in roots, respectively, and increased first and then decreased with the increase in Zn treatment concentrations. Oxalic acid and malic acid were mainly in shoots and accounted for 19.04–31.83% and 36.35–36.96% of the total organic acids in shoots, respectively. The organic acids of xylem sap were mainly oxalic acid and malic acid, accounting for 12.89–25.42% and 48.01–50.86% of the total organic acids in the xylem sap, respectively. Compared to CK, the contents of oxalic acid decreased by 58.21%, 73.21%, and malic acid decreased by 50.34%, 33.48% under 100 mg kg^−1^ and 300 mg kg^−1^ Zn treatment concentrations, respectively.

### 2.5. Correlation of Organic Acid Contents with Cd and Zn Contents in Different Parts of B. juncea

Table 2 shows correlations between Cd and Zn contents in roots and shoots of *B. juncea*. The Cd content in roots was significantly and negatively correlated with acetic acid. Zn content correlated significantly and positively with citric acid and negatively with tartaric and malic acid in shoots.

The correlations between the Cd and Zn contents and the organic acids in the vacuole of *B. juncea* are shown in Table 3. Oxalic and succinic acids were highly negatively correlated with Cd content in the vacuole of roots, while malic, malonic, and succinic acids were significantly positively correlated. The Zn content of leaf vacuoles correlated significantly positively with tartaric acid and acetic acid, and negatively with citric acid.

The correlation between different indicators was tested using stepwise regression analysis. Y is the Cd and Zn contents in the xylem sap, *X*1 is oxalic acid, *X*2 is tartaric acid, *X*3 is malic acid, *X*4 is malonic acid, *X*5 is acetic acid, *X*6 is citric acid, and *X*7 is succinic acid. The regression equation for Cd in xylem sap was

(1) Y=0.004−0.029X1+0.023X4−0.047X6 (P≤0.05,r=0.999,F=288.89)

Thus, the Cd content in the xylem sap of *B. juncea* correlated significantly and negatively with oxalic acid (*X*1) and citric acid (*X*6), and positively with malonic acid (*X*4). The regression equation for Zn in xylem sap was

(2) Y=0.035−0.370X1+0.395X4 (P≤0.05,r=0.981,F=77.60)

Thus, the Zn content in the xylem sap of *B. juncea* significantly correlated positively with malonic acid (*X*4) and negatively with oxalic acid (*X*1).

## 3. Discussion

### 3.1. Heterogeneity of Cd and Zn Translocation and Distribution in B. juncea

This study showed that the Cd and Zn contents in roots were distinctly higher than those in shoots after 20 days of treatment (Table 1). This is consistent with previous findings demonstrating that Cd and Zn accumulate primarily in roots [7,26]. In addition, a significant decrease of translocation factor (TF) was observed as Cd and Zn treatment concentrations increased, a result that had been reported in other plants as well [6,15]. Furthermore, high root retention of Cd and Zn suggests *B. juncea* mainly accumulates Cd and Zn in the roots and prevents their translocation to shoots, which is one of the mechanisms by which plants resist Cd and Zn toxicity [27].

In a previous study, it was found that Cd was usually absorbed by plants through the symplast pathway into the xylem after loading and thus accumulated in the roots due to the presence of the apoplast barrier [28]. In this study, the contents of Cd in the symplast sap were significantly higher than those in the apoplast sap (Figure 1), which had also been reported in another study [15]. This result shows that the symplast pathway accounted for most of the absorption of Cd, while the apoplast pathway also played a role. This was in line with the findings of a previous study suggesting that the apoplast pathway can also influence the uptake of Cd in plants [29]. As a result of this study, Zn contents in the apoplast sap showed an increasing trend with increasing Zn treatment concentrations, and Zn contents in the symplast sap were lower than those in the apoplast sap (Figure 1). This was in agreement with the study of others [4]. This suggests that there are differences in the way that Cd and Zn are taken up by plants.

It has been found that xylem sap composition is usually related to long distance transport in the plant [30]. Our study showed that the Cd content in xylem sap was higher than that in phloem sap under a 40 mg kg^−1^ Cd treatment (Figure 2), which was similar to the results found in another study [18]. The Cd and Zn accumulation in shoots was determined by xylem transport [31]. The Cd contents in xylem sap showed an increasing trend with increasing Cd treatment concentrations; a similar result had been reported in another study as well [15]. However, the Zn contents in xylem sap and phloem sap showed an increasing trend with increasing Zn treatment concentrations (Figure 2), suggesting that Zn can be transported through xylem and phloem in plants. This was in line with the transport of Cd and Zn in the phloem, which has been studied and reported in *Arabidopsis thaliana*, *Arabidopsis halleri*, and *Oryza sative* L. [32,33,34]. It may be transported to the root cell to participate in the synthesis of transporter proteins due to its physiological role [35]. According to these results, Cd is mostly absorbed by the symplast and Zn by the apoplast, and both are then transported via the xylem to the shoots [36]. Further, from root to shoot, xylem transport is the main determinant of Cd and Zn content in shoots [4,15].

Previous studies have shown that plant cell walls, as the first line of defense against heavy metal toxicity in protoplasts, and the vacuole also have a compartmentalizing effect on Cd and Zn that enter the cells [20,21,37]. In this study, at the subcellular level of plants, 55–58% of Cd in roots and 54–81% of Cd in leaves were distributed in the vacuole, and 30–34% of Cd in roots was present in the cell wall (Figure 3), which had been similar results for *Morus alba* L. [21]. The Cd contents in the cell walls of roots and leaves showed an increasing trend with increasing Cd treatment concentrations (Figure 3), which corroborated the cell wall binding and fixation of Cd [37]. However, this study showed that 74–78% of Zn in roots and 66–68% of Zn in stems under Zn treatments were bound to the cell wall, and 41–47% of Zn in leaves was distributed in the vacuole (Figure 4). This was in line with the findings of a previous study, which suggested that Zn in roots and stems was mainly located in cell walls and that Zn in leaves was mainly stored in the vacuole [38]. The excess Zn affected chlorophyll synthesis [39] and was involved in some protein synthesis [40], which may explain the different distribution of Zn in leaves compared to roots and stems.

### 3.2. Effect of Organic Acid Heterogeneity on the Translocation and Distribution of Cd and Zn in B. juncea

According to our study, oxalic and malic acids were present in high concentration in roots and leaves (Figure 5 and Figure 6), suggesting that both are crucial to the accumulation of Cd and Zn in *B. juncea.* And Kang et al. [41] reported that malic acid content was highest in two cultivars of melon (*Cucumis melo* L.). In this study, the organic acid contents in roots decreased with increases in Cd and Zn treatment concentrations, but the organic acid contents in leaves first decreased and then increased (Figure 5). This was in agreement with the results of previous studies [42,43]. This result may be due to the fact that the low Cd or Zn concentration treatment did not affect the normal growth of plants, while under the high concentration treatment, plants alleviated the toxicity of Cd and Zn by secreting organic acids [44,45]. In addition, the vacuole has been reported to be the main site of organic acid storage [45], and Cd and Zn are chelated with organic acids in the vacuole [24]. This study showed that the Cd contents in roots were negatively correlated with oxalic and succinic acids (Table 3), the Zn contents in roots were positively correlated with malic, malonic, and succinic acids, and the accumulation of Zn in leaves was positively correlated with tartaric acid and acetic acid and negatively correlated with citric acid (Table 3), indicating these organic acids are likely chelators involved in Cd and Zn accumulation in the vacuole. 

The metal-chlorate complexes play an important role in the phytoextraction of heavy metals, and their stability affects transportation efficiency directly [46,47,48]. In this study, the oxalic and malic acids were that present in high contents in xylem sap decreased with increasing levels of Cd and Zn treatments (Figure 5 and Figure 6), while the translocation factor of Cd and Zn decreased. This was in line with the findings of previous studies [49,50]. However this suggests that both are crucial to the translocation of Cd and Zn in the xylem sap of *B. juncea.* The results of stepwise regression analysis showed that oxalic acid, malonic acid, and citric acid may be the main influencing factors of Cd and Zn contents in xylem sap. Similarly, the study by Ueno et al. [23] showed that a small portion of Cd could be complexed with citric acid, and Li et al. [51] reported that Cd was associated with *A. halleri* and *O. sativa* xylem sap in the form of citric acid. Notably, different plant species and types of heavy metals may lead to inconsistent results. Therefore, oxalic, malic, and citric acids may be involved in the translocation process of Zn, but further study is needed.

## 4. Materials and Methods

### 4.1. Plant Material and Soil

The *B. juncea* seeds were purchased from Shuyang Zee Yirou E-Commerce Co., Ltd. (Jiangshu, China). The seeds were first disinfected with 10% sodium hypochlorite for 3 min and then washed with deionized water. Then, the cleaned seeds were dried and scattered in the tested soil for germination. Well-grown, uniformly sized seedlings (with 4–6 leaves, 8–10 cm height) were selected for a pot experiment.

The tested soil was purchased from Yunnan Mile Tobacco Materials Co., Ltd. The soil type was substrate soil, the soil pH was 4.49, and organic matter was 431.75 g kg^−1^, available N was 437.50 mg kg^−1^, available P was 81.45 mg kg^−1^, and available K was six 220.17 mg kg^−1^. The total Cd and Zn in the soil were 0.26 and 74.50 mg kg^−1^, respectively. In referring to the “Soil Environmental Quality: Risk Control Standard for Soil Contamination of Agricultural Land” (GB 15618-2018), when the soil pH is less than 5.5, the risk screening values for Cd and Zn in agricultural land are 0.3 mg kg^−1^ and 200 mg kg^−1^, respectively. The Cd and Zn contents of the soil used in this experiment were lower than this value.

### 4.2. Experimental Design

In the experiment, 2 kg of tested soil were put into each pot (leave spaces of 33 cm, 24.5 cm, and 13.5 cm). The Cd concentrations in the experiment were designed to be 20 and 40 mg kg^−1^ (Cd20 and Cd40), the Zn concentrations in the experiment were designed to be 100 and 300 mg kg^−1^ (Zn100 and Zn300), and the control was carried out using test soils without exogenous addition of Cd or Zn (CK). Each treatment was replicated three times. The Cd and Zn treatments were done by preparing CdCl_2_∙2.5 H_2_ O and ZnSO_4_∙7 H_2_ O into solutions and pouring them evenly into the soil, where they were left for 5 days to precipitate Cd and Zn. Then, seedlings that grew in the same condition were selected and cultivated for twenty days during the incubation period. 

After 20 d of treatment, the plants were harvested. The plants were first measured for fresh weight, washed, and divided into root and shoot. A portion of root and shoot plant samples were put in a blast dryer (DHG-9145 A, Shanghai Yiheng Scientific Instruments Co., Ltd., Shanghai, China) at 105 °C for 30 min, and then dried at 70 °C to a constant weight and ground into dry samples for the measurement of the Cd and Zn contents. The other portion of the shoots and roots plant samples were stored in the −80 °C refrigerator for the measurement of other parameters (including Cd and Zn contents in sap, Cd and Zn subcellular distribution, and organic acid contents in various plant parts).

### 4.3. Indicator Measurement Method

#### 4.3.1. Plant Cd and Zn Contents

The Cd and Zn concentrations in plant tissues (roots and shoots) were determined according to the method reported by Wu et al. [15] and then improved by improving the acid digestion system. The dried plant samples (0.1 g) were put into the ablation tank, 3 mL HNO_3_ was added overnight, 2 mL H_2_ O_2_ was added; digest at 160 °C for 4 h until clarification; filter; fix the volume of deionized water was fixed at 50 mL, and the contents of Cd and Zn were measured by flame atomic absorption (Thermo iCE^TM^-3000, Thermo Fisher Scientific, Waltham, MA, USA). The translocation factor of heavy metals in plants was calculated as:

(3) $$\mathrm{Translocation}\;\mathrm{factor}\;(TF)=\frac{\mathrm{Heavy}\;\mathrm{metal}\;\mathrm{concentrations}\;\mathrm{in}\;\mathrm{shoots}}{\mathrm{Heavy}\;\mathrm{metal}\;\mathrm{concentrations}\;\mathrm{in}\;\mathrm{roots}}$$

#### 4.3.2. Extraction Method of Root Apoplast and Symplast Saps

The fresh samples of roots (0.5 g) were soaked twice in 50 mmol L^−1^ MES-Tris buffer (pH 6.5) for 20 min at 0.5 kPa pressure. Then root samples were removed, the surface water was blotted out, and the samples were centrifuged at 4 °C for 15 min at 1500 g to collect the apoplast sap. After collecting apoplast sap, the roots were frozen at −20 °C for 3 days and centrifuged at 4 °C for 15 min at 3000 g to obtain symplast sap I. Add 1 mL ethanol to the roots and homogenize for 3 min. The supernatant was taken after centrifugation, and the procedure was repeated once, and the two supernatants were combined to obtain symplast sap II. The two supernatants were combined to obtain the symplast sap [15].

#### 4.3.3. Extraction Method of Xylem and Phloem Saps

The *B. juncea* was moved to a shaded place, and the stalks were quickly cut at 4 cm from the roots’ base with a blade sterilized by acetone soaking, and the first drops of sap were discarded with skimmed cotton dipped in deionized water, quickly wiping dry the incision attached to the roots and blotting out the bright surface. The overflowing sap was then aspirated cyclically with a 200 µL Eppendorf pipette in 10 mL Eppendorf tubes, with 5 plants per replicate, and collection was stopped after 12 h. The xylem exudate was centrifuged for 10 min at 3000× *g* and the suspension was collected, and stored in 1.5 mL Eppendorf tubes in an ultra-low temperature refrigerator (below −70 °C) for use [12]. The shoots cut during xylem sap collection were washed with deionized water and blotted on the surface with skimmed cotton, inserted into small plastic bottles containing 15 mL of 25 mmol L^−1^ EDTA-Na_2_ solution, placed in a shaded and sealed incubator, maintained at a relative humidity greater than 95%, and the sap of the bast was collected at 20 °C for 24 h [52].

The collected apoplast, symplast, xylem, and phloem saps were fixed in a volume of deionized water of 50 mL, and the Cd and Zn contents were subsequently measured by flame atomic absorption (Thermo iCE^TM^-3000, Thermo Fisher Scientific, Waltham, MA, USA).

#### 4.3.4. Measurement of Subcellular Contents of Cd and Zn in Roots, Stems, and Leaves of *B. juncea*

The extraction assay of subcellular fractions was performed using the previous method [53], where plant roots, stems, and leaves were divided into cell wall fractions (F1), organelle fractions (F2), and vacuole fractions (F3). The experiment used a pre-chilled extraction solution (250 mmol L^−1^ sucrose + 50 mmol L^−1^ Tris-HC1 buffer (pH 7.5) + 1 mmol L^−1^ dithiothreitol). 

The fractions F1 and F2 were placed in the digestion tank; 3 mL HNO_3_ was added overnight; 2 mL H_2_ O_2_ was added; they were digested at 160 °C for 4 h until clarification, filtrated, and fixed to 50 mL, and the Cd and Zn contents were measured by atomic absorption. The fraction F3 was directly measured by flame atomic absorption using the extract as the background control to measure the Cd and Zn contents, and the proportion of Cd and Zn in each fraction was also calculated (Thermo iCE^TM^-3000, Thermo Fisher Scientific, Waltham, MA, USA).

#### 4.3.5. Xylem Sap and Plant Organic Acid Contents of *B. juncea*

A mass of 0.1 g of shoots and roots were weighted, add 2 mL of deionized water; grind to homogenate, and centrifuged at 20,000 r min^−1^ for 20 min, and take as the supernatant. The supernatant was filtrated through a 0.45 μm membrane and saved in a 1.5 mL brown injection bottle for measurement [41,54]. The xylem sap collected in 4.3.3 was similarly filtrated through a 0.45 μm membrane and saved in a 1.5 mL brown injection bottle for measurement.

Each organic acid was measured by an external standard method using HPLC (Thermo HPLC-Ultimate 3000, Thermo Fisher Scientific, Waltham, MA, USA). A 150 × 4.6 mm C18 column (5 μm grain size) was used for the separation of chromatography. The column temperature was set at 30 °C. The mobile phase A was 0.01 mol L^−1^ KH_2_ PO_4_ (pH 2.75) and phase B was methanol, and the elution was carried out with 95% A + 5% B for 0–10 min. The flow rate was set to 1.0 mL min^−1^. The injection volume is 20 μL, and the detection is performed at 210 nm in UV wavelength [41].

### 4.4. Statistical Analysis

The one-way analysis of variance (ANOVA) was used to determine the significant differences among treatments, which were performed with SPSS 21.0 (SPSS Inc., Chicago, IL, USA). The Duncan’s test was used to compare the means of the control and treatment groups at a significance level of *p ≤* 0.05. The translocation factor (TF) was developed to assess Cd and Zn for translocation in plants. The ratio of the Cd or Zn content in shoots to that in roots is known as the TF. 

The figures were graphed using Origin Pro 2021 (OriginLab, Northampton, MA, USA), and stepwise regression analysis was carried out using SPSS 21.0.

## 5. Conclusions

In this research, it was observed that Cd absorbed by *B. juncea* was dominated by the symplast pathway and Zn by the apoplast pathway. Cd and Zn are mainly accumulated in the root system and can be transported mainly upward through the xylem. Results on the subcellular distribution of *B. juncea* revealed that Cd was mainly located in the vacuole and Zn was mainly located in the cell wall, respectively. Furthermore, oxalic and malic acids were present in high concentrations in roots, leaves, and xylem sap. According to a correlation analysis of Cd, Zn, and organic acids in the vacuole, there was a negative correlation between Cd content and oxalic acid and succinic acid contents, and a positive correlation was found between Zn content and malic and acetic acid contents. Based on stepwise regression analysis, we found that the contents of Cd were negatively related to the contents of oxalic and citric acid in xylem sap, and the contents of Zn were negatively related to the contents of oxalic acid in xylem sap. These results show that oxalic, malic, and citric acids are important factors affecting the migration and distribution of Cd and Zn. Therefore, this experiment can provide a basis for studying the translocation and molecular mechanism of oxalic, malic, and citric acid affecting Cd and Zn in the xylem and vacuole of *B. juncea*.

## Figures and Tables

**Table 1:** Biomass and Cd and Zn contents in shoots and roots of *B. juncea*.

Heavy Metal	Treatment Concentrations/mg kg^−1^	Shoots		Roots		TF
		Biomass/g plant^−1^	Contents/mg kg^−1^	Biomass/g plant^−1^	Contents/mg kg^−1^	
Cd	0	4.50 ± 0.17 a	0.49 ± 0.21 b	1.68 ± 0.08 b	0.66 ± 0.15 c	0.90
	20	4.18 ± 0.16 b	3.27 ± 0.60 a	1.65 ± 0.06 b	11.55 ± 3.05 b	0.28
	40	4.54 ± 0.09 a	3.80 ± 0.62 a	2.38 ± 0.09 a	31.83 ± 6.13 a	0.11
	0	4.50 ± 0.17 a	21.85 ± 4.49 b	1.68 ± 0.08 a	30.30 ± 3.32 c	0.72
Zn	100	4.06 ± 0.13 b	51.30 ± 9.51 a	1.77 ± 0.10 a	106.8 ± 12.65 b	0.48
	300	3.18 ± 0.13 c	53.55 ± 8.98 a	0.91 ± 0.08 b	211.43 ± 10.56 a	0.25

Note: TF was translocation factor. The different lowercase letters indicate differences in the same column under Cd and Zn treatments at the *p* ≤ 0.05 level of significance. The same as below.

**Table 2:** The correlation between organic acid contents and Cd, Zn contents in roots and shoots of *B. juncea*.

Parts	Heavy Metal	Organic Acid						
		Oxalic Acid	Tartaric Acid	Malic Acid	Malonic Acid	Acetic Acid	Citric Acid	Succinic Acid
roots	Cd	−0.758	0.536	−0.204	−0.243	−0.820 *	−0.256	−0.657
	Zn	−0.307	0.025	−0.588	−0.435	0.141	−0.412	−0.866
shoots	Cd	−0.531	0.011	0.210	−0.524	−0.624	0.324	0.134
	Zn	−0.262	−0.796 *	−0.860 *	−0.430	−0.742	0.797 *	−0.091

Note: * indicates significant correlation (*p ≤* 0.05).

**Table 3:** Correlation between Cd and Zn contents and organic acids concentrations in vacuole of *B. juncea*.

Parts	Heavy Metal	Organic Acid						
		Oxalic Acid	Tartaric Acid	Malic Acid	Malonic Acid	Acetic Acid	Citric Acid	Succinic Acid
Vacuole of roots	Cd	−0.968 **	−0.032	0.369	0.237	−0.749	−0.653	−0.918 **
	Zn	−0.442	−0.455	0.840 *	0.836 *	0.474	0.673	0.828 *
Vacuole of leaves	Cd	0.020	0.511	0.707	0.003	−0.232	0.342	0.638
	Zn	0.088	0.861 **	0.383	0.584	0.889 **	−0.810 **	0.568

Note: * indicates significant correlation (*p ≤* 0.05), ** indicates highly significant correlation (*p ≤* 0.01).

## Data Availability

The original contributions presented in the study are included in the article. Further inquiries can be directed to the corresponding author/s.
